# Association between age and loneliness in different residential type and gender groups: evidence from China

**DOI:** 10.1186/s12888-023-04525-1

**Published:** 2023-01-17

**Authors:** Yang Wang, Weikang Jiang, Lei Wu

**Affiliations:** 1 Department of Social Work and Social Policy, School of Sociology and Population Studies, Renmin University of China, No. 59, Zhongguancun Street, Haidian District, Beijing, China; 2 School of Public Administration, Xiangtan University, Xiangtan, China

**Keywords:** Loneliness, Age, Residential type, Gender, China

## Abstract

**Background:**

Age has been identified as a prominent predictor of loneliness, although the findings about the relationship between age and loneliness are inconclusive. This study examines the relationship between age and loneliness in the context of China, with a focus on residential and gender differences.

**Methods:**

Data were from the Chinese General Social Survey (CGSS) of 2017. A total of 3899 respondents were included. Loneliness was measured using a three-item Short Loneliness Scale. Age, squared terms of age, residential type, gender, and other socio-demographic characteristics were included in the study. Regression analyses were conducted among the total sample and subgroups of different gender and residential type subgroups, to investigate the association between age and loneliness.

**Results:**

There is a reverse U-shaped tendency between age and loneliness that peaks at the age of 47. This tendency is true of the male subgroup, that peaks at the age of 55, while the female respondents do not share that tendency. The inverted U-shaped distribution holds true for urban but not for rural residents. The female respondents reported a higher level of loneliness than the male. The rural respondents reported higher loneliness than their counterparts.

**Conclusions:**

This study demonstrates that an inverted U-shaped tendency between age and loneliness existed for the entire group, and the male and urban subgroups. Implications for service and practice are proposed based on the empirical findings.

**Supplementary Information:**

The online version contains supplementary material available at 10.1186/s12888-023-04525-1.

## Introduction

Most people have experienced loneliness at some point in life. Loneliness is usually defined as a negative subjective feeling of deficit in social contact, which is often caused by the loss or lack of social relationships [1, 2]. Weiss (1975) specified two types of loneliness: “social loneliness” which refers to the absence of social networks one gets highly engaged in, and “emotional loneliness” which refers to the absence of intimate attachment figs [3]. Loneliness has a detrimental effect on individuals’ well-being, i.e., adverse physical and mental health outcomes [4, 5], and can be related to problem behaviors like suicide [6]. Various factors were found associated with loneliness, including demographic, socioeconomic, health-related, and personality factors [7, 8]. That association, however, needs to be further verified, especially in developing countries where a lack of attention has been paid to it.

Age has been identified as a prominent predictor of loneliness [9]. Considering the available research results, there were two types of relationship between age and loneliness in the available research. First, linear relationships imply “the older, the lonelier” [10, 11]. Second, other studies found non-linear relationships. For example, Luhman & Hawkley (2016) found that the highest level of loneliness was experienced by young adults and the oldest old [12], while other scholars found an inverted U-shaped relationship [13]. Other studies found no linkage between age and loneliness. Therefore, there is no conclusive finding about the relationship between age and loneliness.

The extant literature on that relationship was mostly established in Western countries, lacking a description of the associations between loneliness and age in less industrialized countries like China. China is the world’s most populous country, accounting for 18% of the global population [6]. In past decades, China has experienced great internal rural-to-urban migration [14], as a result of more flexible economic policies and a requirement for more labor in industrial factories than agricultural fields. This has contributed to rapid urbanization [15, 16]. In the past four decades, China’s urban population grew from 17.9 to 63.9% [14]. One study in 2015, which used a nationally representative sample of middle-aged and older Chinese, found that over one-third of respondents in urban areas were rural-to-urban migrants. Correspondingly, over one-third of respondents in rural areas in China were *left-behind* family members [17].

Along with the rapid social changes in China, there is a sharp rise in social problems and disparities in welfare and mental health care provision [18]. The residential type system has become a social boundary distinguishing rural residents from urban residents. Compared with urban residents, rural residents have relatively lower formal social security and other resources, such as medical and educational services [19, 20]. Numerous studies have found that rural residents have higher levels of loneliness. It has been widely reported by rural children who are separated from their migrant parents and suffer more psychological problems [21, 22]. It is also evident that older adults residing in rural areas have an elevated risk for depression [23]. However, the relationship between age and loneliness with different residential types in China is unknown in the extant research.

Two perspectives informed our exploration of the association between age and loneliness among people with different residential types. First, the distribution of loneliness may be inferred from the perspectives of collectivism and individualism [24, 25]. According to the WHO mental health surveys, mental health problems such as depression and anxiety had an overall lower prevalence among the populations in collectivist countries [26]. As for loneliness, it was believed that compared to residents in collectivistic countries, those residing in individualistic countries tended to have a lower level of loneliness [27, 28]. In the context of China, based on the general observation, it is well acknowledged that the components of individualism are more adaptive to the urban environment, while components of collectivism are more adaptive to the rural environment [29, 30]. This implies that urban and rural residents will have different associations of loneliness with age.

Second, the transition of individuals’ social and behavioral features can occur with the process of urbanization. Along with China’s unprecedented urbanization, the effect of the cultural mismatch between rural and urban areas is evident. The transformation in people’s living environments can lead to shifts in socialization and cultural values. For example, residents who are exposed to the urban and individualistic environment but still hold rural and collectivistic cultural values might find themselves maladaptive to the environment [31]. Accordingly, the rural-to-urban migrants would experience loneliness in the transition.

Recent studies demonstrated that loneliness was more common in urban areas [32, 33]. Thus, it is rational to assume that some urbanized Chinese of different ages are at greater risk of loneliness than their rural counterparts. For example, young adults who lived alone in cities for higher education or jobs experienced high living pressure, simple social connections and rare communication with others [34, 35]. It could take them more effort to rebuild their social networks, and they were found to have a higher level of loneliness than their rural counterparts [36, 37]. Residents living in rural areas experience more stable social networks to counter loneliness. This contrasts with urban residents, where the individualistic environment caused them to be more vulnerable to loneliness. Many urban residents also experience a cultural value mismatch. It is reasonable to assume that a difference can exist in the age distributions of loneliness between Chinese residing in urban and rural areas. It is meaningful to investigate the potential difference and target the specific groups of the population that deserve more attention and assistance in addressing loneliness.

The association between gender and loneliness is often assumed. Some scholars posit that women are more vulnerable to loneliness than men [38, 39]. This is because women live longer, and they are more likely to be spousal caregivers and experience widowhood. It is also known women might have higher expectations of social contact [40, 41]. Nevertheless, other scholars argue that men tend to spend more time alone from adolescence, and negative life events in late adulthood—such as losing loved ones—had a more detrimental impact, which increases loneliness [42]. The empirical results are inconclusive about whether women are lonelier than men [43, 44]. Therefore, despite the different development trajectories and characteristics of men and women, their gender roles in relationships are socialized to some extent [45], so the impact of gender could be moderated by other factors, such as age and social context [43]. Additionally, previous research mainly focused on gender comparison in particular developmental periods, for example, in young [12] or old age [40, 41], while there is a lack of lifespan perspective. Therefore, it is meaningful to test the potential gender difference in loneliness without a fixed a-priori hypothesis and explore the potential gender difference in the associations of loneliness with age.

To summarize, few studies have explored the age distribution of loneliness in China. The context in China warrants an exploration of the relationship between age and loneliness on the condition of residential type and gender. To address this gap, this study employs a nationally representative dataset to answer the following three questions: First, what is the relationship between age and loneliness among the Chinese population? Specifically, is there a linear or curved correlation? If a curved correlation exists, will it be a U-shape or an inverted U-shape? At what age does the highest level of loneliness appear? Second, could the age distribution of loneliness vary by residential type? Third, could the age distribution of loneliness vary by gender?

## Method

### Data and sample

The data for this study were drawn from the open-access 2017 Chinese General Social Survey (CGSS, http://cgss.ruc.edu.cn/info/1014/1019.htm) to examine the relationship between age and loneliness in terms of residential type and gender. CGSS is of the earliest, ongoing, and most comprehensive large-scale social survey projects at the national level in China—initially launched in 2003. With a multi-staged stratified sampling scheme, CGSS2017 covers 12,582 households from 480 community-level units in 28 provincial administrative regions, with the aim of capturing the long-term trends of social change in China. Comparison with the age structure in the China 2020 Census data revealed that CGSS2017 was representative in terms of age distribution (see Additional file 1 Appendix 1). In addition, CGSS2017 is also part of the International Social Survey Program (ISSP), so it can be compared with other participating countries. CGSS2017 is the most recent wave in which the key variable of loneliness is included for the first time [46]. According to the design of the survey, only 4199 participants were asked to report their loneliness. After excluding 300 cases with missing values, a final sample with 3899 participants was obtained.

### Measures and variables

#### Loneliness

In CGSS2017, loneliness was measured by the Short Loneliness Scale [46, 47] of three items. Participants were asked, “How often do you feel that you lack companionship?” “How often do you feel left out?” and “How often do you feel isolated from others?” It uses a three-point Likert scale ranging from 1 for “hardly ever”, to 2 for “sometimes”, and 3 for “often”. The total scores on the scale range from 3 to 9, with higher scores representing a greater degree of loneliness. The Chinese version of the Short Loneliness Scale has been used to evaluate the feeling of loneliness among Chinese adults [48]. This study had a Cronbach’s alpha of 0.817.

#### Age

Age was treated as a continuous variable to describe the general trends in loneliness with age. Since there is no conclusive result on the associations of loneliness with age for the Chinese population, age^2^ was also used to capture the peak.

#### Gender

Gender was measured as a dichotomous variable (0 = male, 1 = female).

#### Residential type

The residential type was measured as a dichotomous variable that distinguished rural residents from urban residents (0 = rural, 1 = urban). The self-reported household registration (*hukou*) status of the participants was used to measure their residential type. The *hukou* system was introduced in 1958, as a governmental effort to guide the economic development of different districts [49]. There are two types of *hukou*: agricultural and non-agricultural. People automatically obtain their *hukou* based on where they were born or where they live and cannot change it at will. People living in rural areas are regarded as agricultural *hukou*, and those living in urban districts are non-agricultural *hukou*.

#### Socio-demographic variables

Empirical evidence has shown that marital status, educational level, party membership, health status, health insurance, pension status, income level, and family size may be associated with loneliness [48, 50, 51]; As such, the effects of the participant’s marital status (1 = married, 2 = unmarried), educational level (1 = below secondary, 2 = secondary and above), self-rated health status (1 = very bad/bad, 2 = moderate, 3 = good/very good), health insurance (1 = have, 2 = not) and pension status (1 = have, 2 = not) were controlled in the study. Two other continuous variables—personal annual income (RMB, logged) and family size. We also included membership of the China Communist Party (1 = yes, 2 = no) because this was suggested to relieve the feeling of loneliness [52].

## Results

### Descriptive statistics

We used Software for Statistics and Data Science (STATA) 15 to analyze the data. Table 1 shows the characteristics of the overall sample and each subgroup. The sample consisted of 3899 adults in total, aged between 18 and 103 with an average age of 51.31 years. The sample was primarily made up of married adults (76.61%), with secondary or higher education (65.94%), pension (72.48%), and health insurance (92.25%). The average family size of respondents was 2.86 (SD = 1.58). A logarithm was used to calculate income, and the mean value of logged income was 8.38 (SD = 3.81). Almost half of them reported good health (53.86%). The mean of loneliness was 3.72 (SD = 1.35), indicating the overall low-level loneliness of the respondents.

**Table 1:** Descriptive statistics and bivariate analysis results (*N* = 3899)

	Total M (SD)/*n*(%)	Men M (SD)/*n*(%)	Women M (SD)/*n*(%)	Bivariate results	Urban M (SD)/*n*(%)	Rural M (SD)/*n*(%)	Bivariate results
Loneliness (3–9)	3.72(1.35)	3.66(1.33)	3.76(1.37)	−2.34^**^	3.56(1.18)	3.85(1.47)	6.72^***^
Age (18–103)	51.31(16.72)	51.24(16.69)	51.38(16.76)	−0.25	51.85(17.36)	50.85(16.15)	−1.86^*^
Age squared	2912.32(1742.68)	2903.93(1724.40)	2920.16(1759.98)	−0.29	2989.52(1842.51)	2845.91(1649.53)	−1.87^**^
Female (%)	51.68	–	–		52.19	51.24	
Urban (%)	46.24	45.75	46.7	−0.59	–	–	
Married (%)	76.61	77.23	76.03	−0.88	74.49	78.44	−2.91^***^
Secondary education or above (%)	65.94	72.29	60	8.16^***^	85.08	49.48	−25.22^***^
Party member (%)	11.08	15.66	6.8	−8.89^***^	18.75	4.48	14.52^***^
Income (logged)	8.38(3.81)	9.15(3.18)	7.66(4.20)	12.35^***^	9.50(3.33)	7.41(3.94)	−17.74^***^
Self-rated health				3.48^***^			−8.20^***^
Very bad/bad	21.34	18.95	23.57		14.86	26.91	
Moderate	24.8	24.95	24.67		26.46	23.38	
Good/very good	53.86	56.1	51.76		58.68	49.71	
Family size (1–30)	2.86(1.58)	2.86(1.60)	2.85(1.56)	0.16	2.71(1.48)	2.98(1.65)	5.18^***^
Pension (%)	72.48	73.51	71.51	1.40	81.7	64.55	−12.17^***^
Health insurance (%)	92.25	92.09	92.41	−0.36	93.57	91.13	−2.84^***^
N	3899	1884	2015		1803	2096	

*Notes: M* mean, *SD* standard deviation

*Significance levels:* ^*^p < .05, ^**^p < .01, ^***^p < .001

We employed T-tests to analyze the differences between the subgroups. The age distribution of loneliness in gender groups is reported in Table 1. Fifty-two percent of the respondents were women (51.68%), slightly more than men (48.32%). Women had a significantly lower average income (*t = 12.35, p < 0.001*) and lower educational levels than men (*t = 8.16, p < 0.001*). In comparison with men, women reported a significantly higher level of loneliness (*t = − 2.34, p < 0.01*).

Table 1 also displays the difference between urban and rural residents. Forty-six percent of the respondents were urban residents (46.24%), and the rest were rural residents (53.76%). The rural residents were more likely to have a larger family size (*t = 5.18, p < 0.001*), married status (*t = − 2.91, p < 0.001*), a lower educational level (*t = − 25.22, p < 0.001*), less income (*t = − 17.74, p < 0.001*), and a lower tendency to have pensions (*t = − 12.17, p < 0.001*) than their urban counterparts. The rural residents had a higher average score of loneliness (*M = 3.85, SD = 1.47*) than the urban residents (*M = 3.56, SD = 1.18*), and there existed a significant difference (*t = 6.72, p < 0.001*).

### Predicting loneliness

After controlling for the socio-demographic variables, we performed multiple linear regression models to examine the relationships between loneliness and the predicting variables. The results are summarized in Table 2. The potential difference in the effect of age on loneliness by gender and residential type was discussed in the research. We employed the Utest command in STATA to examine the possible U-shaped (or inverse U-shaped) relationships between age and loneliness [53].

**Table 2:** Regression analysis results (*N* = 3899)

	Model1	Model2	Model3	Model4	Model5	Model6
	b (SE)	b (SE)	b (SE)	b (SE)	b (SE)	b (SE)
Age	−0.008	0.016^*^	0.022+	0.012	0.008	0.020+
	(0.007)	(0.008)	(0.012)	(0.011)	(0.013)	(0.010)
Age^2^	0.0001+	−0.0001^*^	−0.0001+	−0.0001	− 0.0001	−0.0001^*^
	(0.000)	(0.000)	(0.000)	(0.000)	(0.000)	(0.000)
Female		0.037			0.048	0.043
		(0.043)			(0.066)	(0.055)
Unmarried		0.464^***^	0.631^***^	0.358^***^	0.466^***^	0.460^***^
		(0.057)	(0.084)	(0.079)	(0.088)	(0.071)
Urban		−0.168^***^	−0.163^*^	−0.171^*^		
		(0.048)	(0.066)	(0.070)		
Secondary education or above		−0.110^*^	−0.175^*^	− 0.049	−0.141+	0.011
		(0.054)	(0.078)	(0.076)	(0.074)	(0.085)
Not party member		0.046	0.057	0.047	0.222	−0.008
		(0.070)	(0.086)	(0.122)	(0.152)	(0.073)
Income		−0.009	0.010	−0.018^*^	− 0.008	−0.011
		(0.006)	(0.010)	(0.008)	(0.008)	(0.009)
Self-rated health (Very bad/bad)						
Moderate		−0.259^***^	−0.278^**^	−0.247^**^	− 0.187^*^	−0.329^***^
		(0.063)	(0.091)	(0.087)	(0.089)	(0.089)
Good/very good		−0.590^***^	− 0.555^***^	−0.638^***^	− 0.601^***^	−0.586^***^
		(0.058)	(0.084)	(0.080)	(0.081)	(0.083)
Family size		−0.074^***^	− 0.061^**^	−0.088^***^	− 0.093^***^	−0.045^*^
		(0.014)	(0.019)	(0.020)	(0.020)	(0.019)
Pension		−0.141^**^	−0.093	− 0.185^*^	−0.146^*^	− 0.136+
		(0.052)	(0.075)	(0.073)	(0.070)	(0.080)
Health insurance		−0.048	−0.138	0.039	−0.102	0.035
		(0.082)	(0.115)	(0.117)	(0.113)	(0.119)
Constant	3.765^***^	4.184^***^	3.795^***^	4.437^***^	4.257^***^	3.752^***^
	(0.180)	(0.253)	(0.362)	(0.359)	(0.404)	(0.322)
Observations	3899	3899	1884	2015	2096	1803
R-squared	0.004	0.088	0.098	0.085	0.087	0.070
Utest	0.71	1.84^*^	1.69^*^	0.85	0.64	1.75^*^

*Significance levels:* ^*^p < .05, ^**^p < .01, ^***^p < .001

Model 1 involved two predictors of age and age^2^. The result shows that, although age was not correlated with loneliness, there was a significant positive association between age^2^and loneliness (*p < 0.1*).

Model 2 showed that, after controlling for the socio-demographic variables, loneliness was positively associated with age (*β = 0.016, p < 0.05*) and negatively related to age^2^ (*β = − 0.0001, p < 0.05*), indicating a reverse U-shaped tendency between age and loneliness. This suggests that, for Chinese adults, loneliness increased significantly with age in the beginning and declined slowly later, after reaching a peak. A turning point existed in the relationship. Further calculation of the marginal effects of age on loneliness indicated that the value of loneliness increased until the age of 47 years old and then declined with age. Model 2 also indicates that, for the entire sample, higher levels of loneliness were related to unmarried status (*β = 0.464, p < 0.001*) and urban residential type (*β = − 0.168, p < 0.001*), while lower levels of loneliness were related to higher education (*β = − 0.110, p < 0.1*), larger family size (*β = − 0.074, p < 0.001*), and having a pension (*β = − 0.141, p < 0.05*). However, we did not find significant differences in the effect of gender on loneliness (*β = 0.037, p>0.05*).

Models 3–4 demonstrated the potential difference in the effect of age on loneliness by gender groups. Consistent with model 2, age and age^2^were both significantly related to loneliness for men, as shown in model 3. Specifically, age was positively associated with loneliness (*β = 0.022, p < 0.1*), whereas age^2^was negatively associated with loneliness (*β = − 0.0001, p < 0.1*), indicating that there could be an upward-sloping curve. The level of loneliness of the male respondents reached its peak value at the age of 55 and decreased after that, which is different from the whole sample. Concerning the potential confounders, unmarried men had a stronger feeling of loneliness (*β = 0.631, p < 0.01*). Compared to unhealthy individuals, those with good health reported Ia lower level of loneliness (*β = − 0.555, p < 0.01*). In model 4, the coefficient values of age and age^2^ were not significantly different from zero. Thus, there could not exist an inverted U-shaped age–loneliness association for women. In comparison to men, women with a higher income were more likely to have a lower sense of loneliness (*β = − 0.018, p < 0.05*). Being covered by a pension plan could significantly reduce women’s sense of loneliness (*β = − 0.185, p < 0.05*), whereas there was no significant difference for men (*β = − 0.093, p > 0.1*).

Models 5–6 displayed the age-loneliness relationship in the rural and urban subgroups, respectively. As shown in Model 5, both age and age^2^ were not significantly associated with loneliness for the rural residents, indicating that there was not a clear age-loneliness association in the subgroup. As for the control variables, being unmarried was positively associated with loneliness (*β = 0.466, p < 0.001*). Higher education (*β = − 0.141, p < 0.1*), good health (*β = − 0.601, p < 0.001*), larger family size (*β = − 0.093, p < 0.001*), and having a pension plan (*β = − 0.146, p < 0.5*) could reduce the sense of loneliness for the rural residents. In Model 6, a significant effect was observed for age and age^2^ for the urban residents. To be specific, age was positively associated with loneliness (*β = 0.02, p < 0.1*), and age^2^ was negatively associated with loneliness (*β = − 0.0001, p < 0.05*). An inverted U-shaped distribution existed, and the degree of loneliness peaked at age 47 and decreased thereafter.

## Discussion

The findings of the association between age and loneliness supported some previous studies which suggested that there was an inverted U-shaped relationship between age and loneliness [8, 12]. The results showed that the loneliness of the entire sample followed an inverted U-shaped curve. The average score of loneliness of 3.72 and over 70% of the respondents with a score of 3 suggested that, for most of the respondents, loneliness was not a significant problem, which supports previous research [54–56].

The finding of a higher level of loneliness among rural residents was consistent with the assertion that loneliness was prevalent among older adults in rural China [57]. This is because intergenerational emotional cohesion is lowered when the younger generation migrates to urban areas for work [58]. The majority of rural-to-urban migrants still hold rural *hukou* status as required by the *hukou* policy [59]. Epidemiological studies have found that many of them experience high levels of loneliness, resulting from social isolation, discrimination, and workplace injustice [60, 61]. Consequently, respondents who hold rural *hukou* can have an overall higher risk of loneliness compared to their urban counterparts, and that could derive from the change of living arrangements and weakened family cohesion, as well as maladaptation in urban areas [62].

This study also shows that urban residents’ age and loneliness followed an inverted U-shape curve with a peak at the age of 47, which differs from previous studies in industrialized countries, where loneliness is more prevalent in young adults or older adults than middle-age adults [11, 12]. There are two possible explanations for the findings. First, middle-aged people’s desire for social interaction may require higher engagement than younger adults. Middle-aged people may expect network interactions of higher quality or closeness, while the size of the network or romance of the relationship are not the key factors [63]. Therefore, although middle-aged people could master more social resources, their desire for interaction might not be met. The second explanation relates to the Chinese context. Most people experience loneliness at every life stage. Nevertheless, the drivers of loneliness in middle adulthood include shifts in family structure, career progression, and changes in health status. However, these challenges could be more difficult to manage in China than in industrialized countries [64]. Since the social welfare system for children and older adults is still underdeveloped in China, Chinese middle-aged urban residents may bear more responsibility for family care, and that might limit engagements that might meet their desire for social relationships.

In our study, there was no significant difference in the effect of gender on loneliness in the fully adjusted models. This result is similar to a previous meta-analysis [43], which found that men and women had similar levels of loneliness throughout life. However, our study found that men’s loneliness followed an inverted U-shape curve, with the peak value at the age of 55, which differs from another study in which loneliness peaked at the ages of 40 and 80 [8]. The finding provided sound evidence of the peak time of loneliness which was not specified in previous studies. This result could be explained by the persistent drop in Chinese female labor force participation in the past 30 years (from 79 to 43%), reflecting the return of the traditional Chinese gender division of labor—“men are breadwinners and women are homemakers” [65]. Accordingly, being male could evoke anxiety about the bureaucratic process, health management, and pre-retirement, which negatively impact men’s social interaction [66]. Moreover, one empirical research found that the division norm was negatively correlated with husbands’ marriage satisfaction [67], which is another risk factor for men’s loneliness. Therefore, middle-aged men were more vulnerable than men at other life stages.

Lastly, our study supports previous findings that being unmarried, having low education, low self-reported health level, co-residing with fewer families, and having no pension were associated with a greater level of loneliness for the entire sample. However, having a pension plan or not mattered only to the female and rural respondents.

The empirical results suggest several implications. The findings of the overall inverted U-shape curve relationship between age and loneliness offer insights into future research on loneliness. More empirical research is needed to verify the findings of the present study in China. For instance, critical factors of residential type, migration, and the condition of being *left behind* should be examined for their influence on the loneliness of Chinese people. Longitudinal models—including information on social economic status, social integration, coping, and personality with more suitable measurement of loneliness—might help to explain the relationship.

For practice and service implications, although the level of loneliness for the entire population was not high, it is still necessary to prevent and mitigate the harmful effects of loneliness for people at different life stages. Based on the findings of the subgroups, gender and locality equality issues should be stressed in practice and services. For example, age-specific services can be developed for the middle-aged male population, to remove the barrier factors and address their need for social interaction. Further measures should be taken to reduce the disparity in mental health services and social security coverage between rural and urban populations. The newly-migrated groups need community work and activities to build up their sense of belonging in the urban community and to reduce discrimination towards them. Professionals could use social advocacy and psychosocial support to attend to the high-risk population groups, such as the middle-aged urban breadwinners and the *left-behind* elderly in the countryside. A gender-friendly environment is required to raise social awareness of the plight of lonely women with low incomes and no pensions. Societal responses need to be mobilized to fight against loneliness and its detrimental effects on health for the whole society. In closing, this study asks the public, academic, and service providers to address the issue of loneliness for ordinary people in China in an era of rapid social and demographic changes, which are challenging for the public administration and social service sectors.

Several limitations should be considered when interpreting the findings. First, this study employed a cross-sectional design which did not allow an investigation of individuals’ early life events and their adaptive process, making it impossible for this study to investigate the developmental trajectories of loneliness of each subgroup. As the exploration of prolonged loneliness—which is probably true for some subgroups of respondents—could help to better explain their current level of loneliness [68], it is necessary to examine the relationship between age and loneliness with a longitudinal design. Second, there should have been questions in the survey asking respondents for more information about their social economic status (e.g., their employment, the length of time they possessed urban *hukou*, and their social capital), social integration, and coping strategies they use to adapt to the urban environment [69]. Third, a more suitable measurement tool for loneliness is needed for future research. One recent study demonstrated that emotional loneliness among the Chinese could have a better indication of the process of urbanization than social loneliness [48]. Nevertheless, the measurement tools of this study had only three items and could not differentiate between emotional and social loneliness. In fact, CGSS2017 is part of the International Social Survey Program (ISSP). In previous studies [70, 71], this scale demonstrated good applicability and representativeness for measuring loneliness. Future studies are expected to provide further analysis of the relationship between age and dimensions of loneliness. Fourth, there are other well-studied factors associated with loneliness that should be included in future research, such as respondents’ personalities.

Despite the limitations, to the best of our knowledge, this study is the first one that used a nationally representative sample to investigate the loneliness of the Chinese population from young adulthood to late adulthood, instead of just focusing on groups at specific life stages. It treated age as a continuous variable instead of categorizing age into groups, allowing it to be among the first studies to explore the age distribution in loneliness. Moreover, this study considered gender and residential type and enriched the literature on loneliness in China and the social context of the nation.

## Conclusion

This study examined the relationship between age and loneliness, in terms of gender and residential type by using a nationally representative dataset of 3899 adults in China. The respondents reported overall low-level loneliness. The female and rural subgroups had higher levels of loneliness than their counterparts. The difference between men and women was insignificant after controlling for all covariates. The regression results demonstrated that an inverted U-shaped tendency between age and loneliness existed for the entire group, and the male and urban subgroups. That tendency did not apply to the female and rural subgroups.

## Supplementary Information

**Additional file 1:** **Appendix 1.** Age structures in China 2020 Census and CGSS2017.

## Data Availability

All citations identified are in the public domain. The datasets used during the current study are available from the corresponding author upon reasonable request.
