# Supplementary figures and images for: Association between age and loneliness in different residential type and gender groups: evidence from China
Source: BMC Psychiatry. 2023 Jan 17;23:43. doi: 10.1186/s12888-023-04525-1 (PMC9843997; doi:10.1186/s12888-023-04525-1)

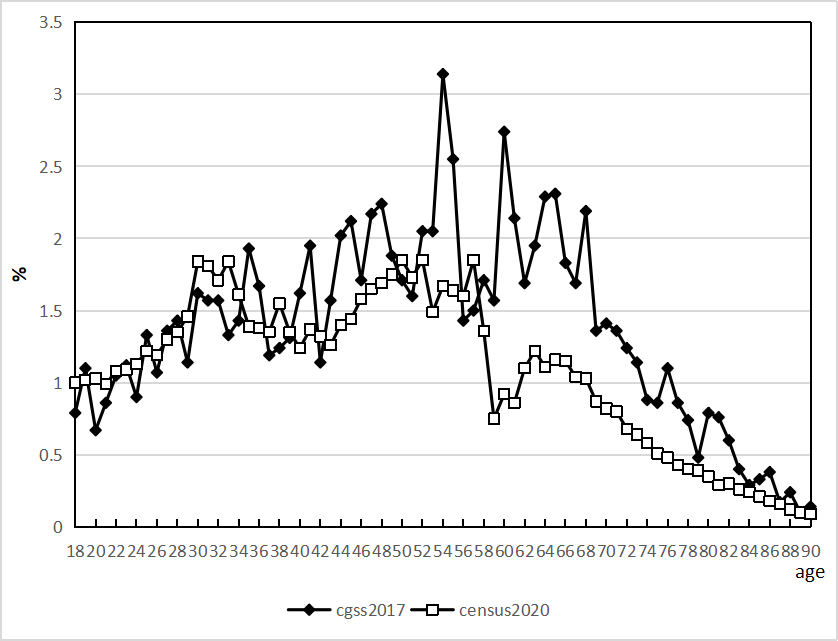


APPENDIX 1. *Age structures in China 2020 Census and CGSS2017*

Supplement: Supplementary file 1 — Additional file 1: Appendix 1. Age structures in China 2020 Census and CGSS2017. [file 12888_2023_4525_MOESM1_ESM.docx]
